# Validation of the Korean version of the avoidance endurance behavior questionnaire in patients with chronic pain

**DOI:** 10.1186/s12955-018-1014-8

**Published:** 2018-09-17

**Authors:** Jeongwi An, Young Hoon Kim, Sungkun Cho

**Affiliations:** 1 Department of Psychology, Chungnam National University, 99 Daehak-ro, Yuseong-gu, Daejeon, Republic of Korea; 2 Department of Anesthesiology and Pain Medicine, Seoul St. Mary’s Hospital, College of Medicine, The Catholic University of Korea, 222, Banpo-daero, Seocho-gu, Seoul, Republic of Korea

**Keywords:** Avoidance behavior, Endurance behavior, Chronic pain, Validation

## Abstract

**Background:**

The avoidance-endurance model suggests both fear-avoidance responses and endurance-related responses could affect the chronicity of pain. Proper pain intervention requires measuring fear-avoidance responses and endurance-related responses but no Korean language questionnaire has yet been made to measure them. The purpose of this study was to evaluate the validity and reliability of the Korean version of the Avoidance-Endurance Behavior Questionnaire (K-AEQ-Behavior) by adapting the behavioral responses of Avoidance-Endurance Questionnaire into Korean language.

**Methods:**

The K-AEQ-Behavior was forward and backward translated based on the standards for instrument translation. A total of 136 outpatients with chronic pain of a duration exceeding 3 months were recruited from a pain center at a university hospital in Seoul, Korea. Two weeks later, the K-AEQ-Behavior was re-administered to 36 patients for test-retest reliability. Exploratory factor analysis was performed using principle axis factoring. The internal consistency, test-retest reliability, and concurrent validity of the K-AEQ-Behavior were measured by Cronbach’s ⍺, intraclass correlation coefficient, and Pearson correlation coefficient, respectively.

**Results:**

Although the four-factor structure (23 items) was derived in the original study, the two-factor structure of avoidance behavior and endurance behavior (21 items) was derived in the exploratory factor analysis of the Korean version in this study. Other results indicated that K-AEQ-Behavior has good internal consistency, test-retest reliability and concurrent validity.

**Conclusion:**

This study suggests that the K-AEQ-Behavior is a reliable and valid instrument for assessing avoidance behavior and endurance behavior in patients with chronic pain.

## Background

The fear-avoidance model suggests that fear-avoidance response (FAR) plays an important role in the development of chronic pain [1]. According to the model, people even avoid behaviors that can help them recover from pain due to fear of pain, resulting in a vicious cycle of continuing and deepening pain. However, some studies have suggested different pathways via which endurance-related responses (ER) also affect development of chronic pain [2, 3]. Specifically, continuing physical activity despite severe pain increases pain by physical overload. The avoidance-endurance model (AEM) suggests that both FAR and ER should be considered in the chronicity of pain [4, 5].

The primary measure of FAR and ER is the Avoidance-Endurance Questionnaire (AEQ) consisting of cognitive, emotional, and behavior responses [6]. Studies on AEQ confirmed that all responses were divided into FAR and ER and have good internal consistencies [6, 7]. Both FAR and ER have several subscales: for example, in behavior response, FAR consists of avoidance of social activities scale (ASAS) and avoidance of physical activities scale (APAS) and ER consists of humor/distraction scale (HDS) and pain persistence scale (PPS). The FAR scales showed positive correlation, but the ER scales negative correlation, with pain-related variables such as depression, disability, and pain-related beliefs.

Proper pain intervention requires measuring FAR and ER but no Korean language questionnaire has yet been made to measure them. Although the original questionnaire consists of cognitive, emotional, and behavioral responses, this study adapts only behavior response to pain because it directly affects the chronicity of pain, as compared to cognitive and emotional responses [1–3]. Therefore, this study aimed to test the validity and reliability of a Korean version of the Avoidance-Endurance Behavior Questionnaire (K-AEQ-Behavior). As evidence for concurrent validity, we set two hypotheses with reference to prior studies [6, 7]. First, FAR scales have moderate-to-large positive correlation with pain catastrophizing, anxiety, depression and pain intensity and moderate negative correlation with mental and physical functioning. Second, ER scales have low-to-moderate negative correlation with pain catastrophizing, anxiety and depression and low-to-moderate positive correlation with mental and physical functioning and pain intensity.

## Methods

### Participants

A total of 136 outpatients were recruited from a pain center at a university hospital in Seoul, Korea. The inclusion criteria were patients with chronic pain of duration exceeding 3 months. Participants whose K-AEQ-Behavior was completed less than 70% were excluded from the analysis, and the final sample was 107 patients. To assess the test-retest reliability of the K-AEQ-Behavior, 36 participants completed the K-AEQ-Behavior two weeks later. Demographic characteristics of the participants are presented in Table 1. The institutional review board approved the study protocol and all participants were given informed consent before participating.

**Table 1:** Demographic characteristics of the sample

Variable	Sample (*N* = 107)
Age (years)	
M	47.5
SD	13.2
Sex (%)	
Male	42.1
Female	57.9
Marital status (%)	
Married	64.2
Non-married	35.8
Educational status (%)	
≥ High school	94.4
Pain duration (months)	
Median	42
Range	3–480
Most significant pain site(s) (%)	
≥ 2 sites	51.4
Lower back	15
Feet (ankle, toe)	6.5
Others	27.1
Diagnosis (%)^*^	
Complex regional pain syndrome	21.2
Spinal stenosis	19.2
Lumbar herniated intervertebral disc	16.3
Postherpetic neuralgia	7.7
Fibromyalgia	6.7

Note: ^*^Duplicate checks were possible in the diagnosis. The ratio was calculated without regard to duplicate checks

### Translation

The K-AEQ-Behavior was translated and reverse translated based on standards for instrument translation [8, 9]. A Korean-speaking clinical psychologist and professional translator who majored in English translated the behavior parts of AEQ into Korean. Then two English-Korean bilingual professional translators, one majored in psychology and another in English, back-translated it. One of the original authors gave feedback on the integrated back-translated version and it was revised accordingly.

### Measures

The types of behavior responses for pain were assessed by the K-AEQ-Behavior in both mild and severe pain. Only behavior responses for severe pain were used in the analysis. Six variables were used to test concurrent validity. Pain intensity was assessed by an 11-point Numeric Rating Scale [10], anxiety and depression by the Hospital Anxiety and Depression Scale (HADS) [11], pain catastrophizing by the Pain Catastrophizing Scale (PCS) [12], and physical and mental functioning by the Short Form-12 (SF-12) [13]. Korean versions of these measures showed good reliability and validity [14–16].

### Data analyses

For the analyses, SPSS 24.0 and AMOS 24.0 were used. Confirmatory factor analysis was conducted to confirm the adequacy of the original AEQ-Behavior factor structure (ASAS, APAS, HDS, PPS) and showed low model fit: comparative fit index (CFI) = .86, root-mean-square error of approximation (RMSEA) = .10, and non-normed fit index (NNFI) = .84. The same process was conducted with the Spanish version of the AEQ-Behavior factor structure (avoidance of social and physical activities scale, pain persistence/distraction scale, ignoring pain scale, humor scale) and also showed low model fit: CFI = .86, RMSEA = .12, and NNFI = .83. Therefore, exploratory factor analysis (EFA) was performed using principle axis factoring with direct oblique. To determine the number of factors suitable for analysis, eigenvalues, plot scree test and parallel analysis were considered. Internal consistency and test-retest reliability were measured by Cronbach’s ⍺ and intraclass correlation coefficient(ICC). In order to verify the concurrent validity of the K-AEQ-Behavior, Pearson correlation coefficient between the scales was performed. As evaluation criterion, *r* < .30 was considered as low correlation, .30 < *r* < .70 as medium correlation, and *r* > .70 as high correlation [17].

## Results

### Factor structure

In EFA, the Kaiser-Meyer-Olkin (KMO) measure of sampling adequacy (.89) and Bartlett’s test of Sphericity (χ2 = 1691.29; *p* < .001) showed that the items were appropriate for EFA. Two factors were extracted considering eigenvalues, plot scree test and parallel analysis. On the second principle axis factoring forced to two-factor solution, items 4 and 5 showed loadings less than .40 in both factors and so were excluded. The final K-AEQ-Behavior was determined as 2 factors with 21 items (Table 2). The 2-factor model accounted for 54.5% of the total variance. One factor included all the FAR items and one ER item of the original AEQ-Behavior, and the other factors included all but three items of the original AEQ-Behavior’s ER items. Thus, these factors were named as avoidance behavior (AB) and endurance behavior (EB).

**Table 2:** Factor loading and descriptive statistics for subscale of K-AEQ-Behavior (N = 107)

Item content		Factor 1	Factor 2	M	SD
8	I cancel a visit to an event.	**.92**	.01	4.03	2.16
7	I cancel private appointments.	**.91**	.00	4.07	2.19
2	I avoid visiting my friends.	**.90**	.05	4.16	2.19
14	I break off a meeting with friends.	**.87**	−.11	3.66	2.17
21	I avoid other people’s company.	**.86**	.08	3.36	2.25
18	I call my guests to cancel an invitation.	**.75**	−.07	3.17	2.27
10	I avoid doing sports.	**.72**	−.03	4.61	1.69
9	I avoid physically strenuous activities.	**.67**	−.16	4.87	1.63
1	I stop doing physically demanding activities.	**.65**	−.27	4.50	1.78
20	I hand over strenuous activities.	**.64**	.05	3.06	2.09
6	I clench my teeth.	**.62**	.21	3.47	2.09
3	I take a rest.	**.50**	−.25	4.79	1.63
23	I distract myself by doing little jobs at home.	−.07	**.79**	2.69	2.16
15	I tell myself: “I don’t have time for this right now!”	.20	**.74**	1.83	1.97
22	I distract myself with physical activity.	−.06	**.71**	2.92	2.10
13	I laugh heartily anyway.	−.28	**.54**	1.65	1.78
16	I take it with a laugh.	−.33	**.53**	1.89	1.89
12	I keep my appointments even though I don’t feel up to it.	−.19	**.52**	2.56	2.15
19	I carry on doing what I am doing no matter what.	−.35	**.50**	2.77	2.04
17	I let my family persuade me into things, even I don’t feel like it.	−.35	**.49**	2.48	2.02
11	I say to myself: “Don’t make such a fuss!”.	.23	**.43**	2.47	2.09
	Eigenvalue	9.23	3.09		
	Variance (%)	43.94	14.69		

Note: Bold number indicates salient factor loading (>.40)

### Reliability

The internal consistencies for AB and EB were Cronbach’s ⍺ = .94 and .85, respectively; test-retest reliability, ICC = .83 and .50 respectively; the standard error of measurement, 7.46 and 8.91, respectively; the minimal detectable change (MDC), 20.67 and 24.70, respectively; and the limits of agreement, − 15.77 to 28.54 and − 28.77 to 18.85, respectively.

### Concurrent validity

Descriptive statistics of the pain outcome variables and their correlations are presented in Table 3. AB showed low-to-moderate positive correlation with pain intensity, anxiety, depression, and pain catastrophizing, but moderate negative correlation with physical and mental functioning, whereas EB showed low negative correlation with depression, but low positive correlation with physical functioning and mental functioning.

**Table 3:** Correlations between the K-AEQ-Behavior subscale and outcome variables

	AB	EB	M	SD
AB	1.00	−.45^***^	47.75	19.08
EB	−.45^***^	1.00	21.27	12.39
Pain intensity	.24^*^	.00	5.50	2.12
Pain catastrophizing	.53^***^	−.13	26.42	14.16
Anxiety	.60^***^	−.18	9.92	5.12
Depression	.55^***^	−.24^*^	12.34	4.24
Physical functioning	−.65^***^	.28^**^	56.18	16.12
Mental functioning	−.67^***^	.28^**^	56.72	19.95

Note: *AB* avoidance behavior; *EB* endurance behavior. ^*^p < .05, ^**^p < .01, ^***^p < .001

## Discussion

The K-AEQ-Behavior appears to be an appropriate measure of behavior responses to pain. The K-AEQ-Behavior showed a two-factor model consisting of AB and EB. AB and EB items correspond to the FAR and ER items of the original questionnaire, respectively, but item 6 (I clench my teeth), which was tied to ER in the original study [6], was tied to AB in this study. It could be due to cultural difference: when Korean people have pain, the expression “I clench my teeth” means to be tolerated rather than to do something despite having pain in Korea [18]. In the original study, FAR consists of ASAS and APAS and ER consist of HDS and PPS [6]. However, there were no subscales in both AB and EB in the present study.

The internal consistency of AB and EB and the test-retest reliability of AB were good (ICC = .83). However, EB showed relatively low test-retest reliability (ICC = .50), possibly due to the low EB score of the study sample at first examination. According to AEM, a high level of FAR or ER is relatively time stable [5]. In this study, patients showed high AB scores and low EB scores (AB = 47.52, EB = 21.96), which may have affected the test-retest reliability. Therefore, it should not be simply determined that the test-retest reliability of ER is low. The MDC scores for AB and EB were 20.67 and 24.70, respectively, at the 95% confidence level. Changes beyond these scores would not be considered as measurement error.

As expected, AB and EB correlated with pain outcome variables in opposite directions. Variables that show positive correlation with AB have negative correlation with EB and vice versa. These results are consistent with two previous studies [6, 7]. However, correlations between EB and pain intensity, pain catastrophizing and anxiety were not significant, which was attributed to the sample characteristics. The sample was recruited from a pain center in a university hospital where patients with severe pain such as complex regional pain syndrome and fibromyalgia visit. Severe pain renders EB performance almost impossible in spite of pain as AEM suggested [19, 20]. Further research is needed to identify how AB and EB correlate with pain outcome variables in various pain intensity groups.

This study had some limitations. First, since the samples were recruited in a pain center of a university hospital, the study findings may not be generalizable to all pain patients. Second, only behavior responses for severe pain were used in this study because we aimed to predict pain outcomes such as pain intensity and disability. Further studies need to investigate the short-term negative or positive effects of AB and EB on cognitive function or emotion when patients experience mild pain. Third, this study cannot identify the pathway that AB and EB lead to intensified pain as AEM suggests, because a cross-sectional design was used. Despite these limitations, this study was the first to validate a measure that discriminates the behavior of pain patients by AB and EB. The study results are expected to contribute to different interventions based on behavioral responses seen by patients.

## Conclusion

The results suggest that the Korean version AEQ-Behavior is a reliable and valid instrument for assessing avoidance behavior and endurance behavior in patients with chronic pain. Considering that the behavior responses to pain affect pain, the K-AEQ-Behavior is expected to provide useful information if used in pain intervention. On the other hand, for generalization to pain patients, additional studies will be needed with a larger sample size and patients with a range of pain intensities.

## Abbreviations

AB: Avoidance Behavior
AEM: Avoidance-Endurance Model
AEQ: Avoidance-Endurance Questionnaire
APAS: Avoidance of Physical Activities Scale
ASAS: Avoidance of Social Activities Scale
CFI: Comparative Fit Index
EB: Endurance Behavior
EFA: Exploratory Factor Analysis
ER: Endurance-related Responses
FAR: Fear-Avoidance Response;
HADS: Hospital Anxiety and Depression Scale
HDS: Humor/Distraction Scale
ICC: Intraclass Correlation Coefficient
K-AEQ-Behavior: Korean version of the Avoidance-Endurance Behavior Questionnaire
KMO: Kaiser-Meyer-Olkin
MDC: Minimal Detectable Change
NNFI: Non-Normed Fit Index
PCS: Pain Catastrophizing Scale
PPS: Pain Persistence Scale
RMSEA: Root-Mean-Square Error of Approximation
SF-12: Short Form-12
